# Encapsulation of *Trichoderma harzianum* Preserves Enzymatic Activity and Enhances the Potential for Biological Control

**DOI:** 10.3389/fbioe.2020.00225

**Published:** 2020-03-25

**Authors:** Cintia Rodrigues Maruyama, Natália Bilesky-José, Renata de Lima, Leonardo Fernandes Fraceto

**Affiliations:** ^1^Environmental Nanotechnology Laboratory, Institute of Science and Technology of Sorocaba, São Paulo State University (UNESP), Sorocaba, Brazil; ^2^Laboratory of Bioactivity Assessment and Toxicology of Nanomaterials, University of Sorocaba, Sorocaba, Brazil

**Keywords:** microencapsulation, *Trichoderma harzianum*, biological control, photostability, polymeric microparticles

## Abstract

*Trichoderma harzianum* is a biological control agent used against phytopathogens and biostimulation in agriculture. However, its efficacy can be affected by biotic and abiotic factors, and microencapsulation has been used to maximize the efficacy. The objective was to develop polymeric microparticles to encapsulate *T. harzianum*, to perform physicochemical characterization to evaluate its stability, to evaluate effects on the soil microbiota, antifungal activity *in vitro* and enzymatic activity. Size distribution of wet and dry microparticles was 2000 and 800 μm, respectively. Scanning electron microscopy showed spherical morphology and encapsulation of *T. harzianum*. Photostability assays showed that encapsulation protected the fungus against ultraviolet radiation. The evaluation of the microbiota showed that the proportion of denitrifying bacteria increased when compared to the control. The *T. harzianum* encapsulation showed an improvement in the chitinolytic and cellulosic activity. *In vitro* tests showed that encapsulated fungus were able to provide a greater control of *S. sclerotiorum*.

## Introduction

For many years, the control of pests and diseases in agriculture has been achieved using synthetic chemicals. However, this has also led to a number of problems for human health, environmental contamination, biodiversity decrease, and pathogen resistance (Zhu et al., 2018). It is therefore desirable to use other safer means of controlling pests and diseases that do not cause adverse effects on human health and the environment. One of such means is through biological control of pests and diseases. The use of biological control agents offers a more sustainable approach that can also address a number of problems associated with the use of agrochemicals in the field (Pazini and Galli, 2011; Li et al., 2012).

*Trichoderma harzianum* is a strong candidate among the various biological control agents. This saprophytic fungus is found worldwide, mainly in the soil (Schuster and Schmoll, 2010). Several species of *Trichoderma*, especially *T. harzianum*, are used in agriculture for their ability to produce large amounts of enzymes and secondary metabolites for the control of phytopathogens (Błaszczyk et al., 2014). The mechanism of action of *Trichoderma* involves mycoparasitism, the production of antimicrobial compounds (Howell, 2003), certain enzymes (Elad, 2000), antibiosis, competition and induction of resistance in host plants (Howell, 2003; Keswani et al., 2014). The hydrolytic enzyme complex that the fungi of the genus *Trichoderma* produce are composed of chitinases, β-glucanases, cellulases, and proteases. These enzymes are capable of decomposing the cell wall of phytopathogens, thus allowing hyphae penetration, colonization and onset of myoparasitism (Hermosa et al., 2012; Carvalho et al., 2015). Among these enzymes, chitinases are the most effective for the biological control of various plant pests and diseases (Ihrmark et al., 2010).

However, commercial formulations based on *Trichoderma* contain live spores and therefore require storing under refrigeration or at temperatures below 28°C, while field applications must be performed under conditions of high relative humidity. Furthermore, ultraviolet (UV) light is harmful to the fungus (Paula et al., 2011; Vemmer and Patel, 2013). Therefore, it is necessary to develop methodologies that can increase bioavailability of this biological control organism in the field. One possible way is to use microencapsulation techniques (Lewis and Papavizas, 1985; Knudsen, 1990; Fraceto et al., 2018). Formulations composed of polymeric microparticles have attracted considerable research interest due to desirable characteristics such as biocompatibility and biodegradability (Poletto et al., 2008b, a; Huang et al., 2009). One of the most advantageous techniques for the production of microparticles is ionic gelation, since it is easy to perform and avoids the use of organic solvents (Rodrigues et al., 2012). Microencapsulation provides a protective barrier around the biological control organism, so that detrimental external factors such as pH, humidity, and ultraviolet radiation do not impair its action (Paulo and Santos, 2017; Locatelli et al., 2018).

Our search of literature has found studies on microencapsulation of *T. harzianum* in polymeric microparticles. In addition, many of these studies have also reported characterization of the microparticles and viability of the fungus after encapsulation (Table 1).

**TABLE 1:** Representative list of published papers in literature about microencapsulation and *T. harzianum* as well as the mode of preparation, used specie and key results.

**Polymeric material**	**Mode of preparation**	**Specie**	**Key results**	**References**
Alginate	Ionic gelification	*Trichoderma* sp.	Characterization: SEM, FTIR, DSC, and DTA Shelf life: 10^6^ CFU/g after 14 months	Locatelli et al., 2018
Maltodextrina and Arabic gum	Spray dryer	*T. harzianum*	Characterization: SEM Shelf life: 40% after 8 weeks	Muñoz-Celaya et al., 2012
Alginate	Extrusion	*T. harzianum*	Characterization: SEM, FTIR e TGA Shelf life: 3 months	Adzmi et al., 2012
Alginate	Emulsion/internal gelation and dripping	*T. harzianum*	UV radiation: resistance after encapsulation Shelf life: 70% after 2 years Biological activity: Antagonism against 3 phytopathogenic fungi	Mancera-López et al., 2018
Chitosan and alginate	Ionic gelification	*Trichoderma viride* and copper	Characterization: Size, FTIR Release: Fickian diffusion	Vincekovic et al., 2016
Alginate		*Trichoderma viride*	Characterization: SEM, FM, CLSM, and FTIR Release: Fickian diffusion Loading efficiency: the increase in calcium concentration decrease loading efficiency	citealpBR28

*SEM (scanning electron microscopy); FTIR (Fourier-transform infrared spectroscopy); DSC (differential scanning calorimeter); DTA (differential thermal analysis); TGA (thermogravimetric analysis); CLSM (confocal laser scanning microscopy).*

Most of the studies presented in Table 1 have reported methods of preparation and characterization of the microparticles containing *T. harzianum*, and the conidia shelf life but have not investigated biological activity or enzymatic activity of the encapsulated fungi. In the present study, we have described the method of preparation of *T. harzianum* containing microparticles and their characterization by scanning electron microscopy (SEM), Fourier-transform infrared spectroscopy (FTIR), and differential scanning calorimetry (DSC). Also, fungal viability assays were performed through study of the shelf life and the effect of UV light. In addition, we have been investigated the effects of the microparticles on soil microbiota as well as enzymatic activity and *in vitro* inhibition of *Sclerotinia sclerotiorum*. These aspects provide new information on the efficacy of microencapsulated *T. harzianum* as a biological control agent against agricultural pests.

## Materials and Methods

### Preparation of Calcium Alginate Microparticles

The microparticles were prepared using the ionic gelation method, which involved dropping a suspension of sodium alginate (ALG – Sigma-Aldrich) containing the organism into a solution of calcium chloride (CaCl_2_ – Synth) (dos Santos et al., 2015). A 2% ALG solution was prepared, followed by addition of the fungus powder (2%, w:v – Ecotrich-Ballagro) and agitated for 1 h to obtain a homogeneous mixture. The alginate solution containing fungus (1:1 v:v) was then dropped into a 0.1 mol L^–1^ solution of CaCl_2_ while agitating on a magnetic stirrer. The microparticles formed were stored at 4°C in a sterile glass vessel. The mean initial quantity of spores in the microparticles containing the encapsulated fungus was estimated at 1 × 10^10^ spores mL^–1^. An aliquot of the particles was also dried in an oven at 27°C to 5% moisture level to compare with wet microparticles.

### Characterization of the Microparticles

#### Size Distribution

The size distributions of the wet and dry calcium alginate microparticles were determined using ImageJ software. Around 500 microparticles were deposited on a surface and analysis was made of individual particles. The size distribution of the dry microparticles was determined after drying in an oven at 27°C for 24 h, with the counting performed in the same manner as for the wet microparticles.

#### Scanning Electron Microscopy

The external and internal morphology of the microparticles was examined by SEM. The samples were dried in an oven at 27°C for 24 h prior to depositing on supports and spray-coating with colloidal gold. The internal surfaces of the microparticles were analyzed after cutting the microparticles using a scalpel. The images were acquired using a JEOL Model JSM-6010LA electron microscope, operated at 3 kV, at the Materials Characterization Multiuser Laboratory of UNESP – Sorocaba.

#### Differential Scanning Calorimetry

Differential scanning calorimetry was used to investigate components of the microparticles, the *T. harzianum* fungus, and the microparticles with or without the encapsulated fungus. The microparticles with or without the encapsulated fungus were dried in an oven, at 27°C for 24 h. The DSC procedure was performed by placing the dry microparticles in aluminum containers and heating from 10 to 400°C, under a 50 mL min^–1^ flow of nitrogen.

#### Fourier-Transform Infrared Spectroscopy

Fourier-transform infrared spectroscopy was used to evaluate the interaction between the fungus and the microparticles. An Agilent FTIR spectrophotometer was used in the range from 4000 to 400 cm^–1^, with 32 scans per sample and resolution of 8 cm^–1^.

### Effect of the Microparticles on Soil Microbiota

The soil used in this analysis (vegetable soil, purchased from agricultural stores is composed by aluminum: 0.832 mg/cm^2^, calcium: 1.675 mg/cm^2^, chlorine: 0.174 mg/cm^2^, iron: 5.654 mg/cm^2^, potassium: 0.454 mg/cm^2^, magnesium:0.252 mg/cm^2^, phosphorus: 0.004 mg/cm^2^, silicon: 15.007 mg/cm^2^, sulfur: 0.049 mg/cm^2^, titanium: 0.755 mg/cm^2^, chromium: 0.004 mg/cm^2^, manganese: 0.029 mg/cm^2^, zinc: 0.007 mg/cm^2^, strontium: 0.008 mg/cm^2^, yttrium: 0.002 mg/cm^2^, zirconium: 0.087 mg/cm^2^, arsenic: 0.000 mg/cm^2^, and bromine: 0.000 mg/cm^2^) was left at ambient temperature in the dark for 3 days and was then homogenized by passing through a sieve (mesh 18). A 10 g portion of the soil was added to a 50 mL tube (area of 7 cm^2^), which was placed in a microcosm. In order to ensure soil conditions similar to those found in the field, ammonium sulfate (Synth) was added at 100 mg N kg^–1^. Deionized water was then added until the soil reached 60% humidity and the tubes were left in the dark at 20°C (Hjelmsø et al., 2014). Subsequently, the soils were exposed to concentrations equivalent to 250 and 2500 g ha^–1^ of sodium alginate, *T. harzianum*, the fungus encapsulated in dry or wet calcium alginate microparticles, dry and wet calcium alginate microparticles, and the negative control (water). After 30 days of exposure, DNA was extracted from the microbiota using the Power Soil^®^ DNA Isolation Kit (MoBio Laboratories, Inc.). The extracted genetic material was quantified by fluorescence (Qubit 3.0 fluorometer), using the Qubit^®^ dsDNA BR Assay Kit (Invitrogen). After quantification, the extractions were diluted to a final total DNA concentration of 1000 ng mL^–1^.

The specific primers used for the molecular evaluation of the soil microbiota were the bacterial 16S rRNA, *nifH*, *narG, nirk*, *nirS*, *norB*, and *nosZ* genes responsible for the production of enzymes linked to the nitrogen cycle (Jung et al., 2012).

For the qPCR reactions, the mixtures were prepared in a final volume of 25 μL containing 12.5 μL of Planium^®^ SYBR^®^ Green qPCR SuperMix-UDG with ROX (Invitrogen), 1 μL of each primer (sense and anti-sense), 100 ng of DNA template, and ultrapure water.

The conditions used for the amplification were as described by Jung et al. (2011), with initial denaturation at 95°C for 3 min, followed by 40 cycles of 95°C for 45 s, 60°C for 45 s, and 72°C for 45 s. The fluorescence was measured at the end of each incubation at 60°C. The analysis employed a calibration curve constructed using serial dilutions of the DNA (1:1, 1:10, 1:100, and 1:1000, v:v). The slope of the curve was used to determine the mean efficiency of the amplifications.

The results for the relative quantification of the samples were provided by the equipment, based on calculation of 2^–ΔΔCt^, where ΔΔCt is the difference between ΔCt for the sample gene and ΔCt for the control gene (Eq. 1), enabling comparison between the control and the material tested.

(1) Δ⁢Δ⁢C⁢t=C⁢t⁢(sample⁢gene)-C⁢t⁢(control⁢gene)

### Quantification of Spores

The quantification of spores was performed by two methods: CFU g^–1^ (colony-forming units per gram of sample) and using a Neubauer chamber. The wet and dry microparticles were evaluated at the start and during storage for up to 120 days at different temperatures (5 and 30°C). For counting the colonies in both microparticles (dried and wet), it was necessary to dilute the microparticles so that the spores could be counted. The dilution was done in 2% sodium citrate (Synth) solution for 20 min, under magnetic stirring (dos Santos et al., 2015).

For CFU g^–1^ analysis, the microparticles were serially diluted in saline solution [0.85% w/v NaCl (Synth)] containing Tween 80 (0.1%). Aliquots of 0.5 mL were plated onto potato dextrose agar (PDA – Sigma-Aldrich) containing Triton X-100 (0.25 g L^–1^ – LGC Biotechnology). The plates were incubated at 28 ± 2°C for 18 h, followed by counting the colonies. The total spores were counted in a Neubauer chamber and the resulting concentrations were expressed as the number of total spores per mL. An aliquot (10 μL) of the diluted microparticles was placed in the Neubauer chamber, with counting of 5 quadrants of each field (fields 1 and 2). The number of spores per mL was calculated for each field and the values were averaged using Eq. 2.

(2) $$\mathrm{Total}\,\mathrm{of}\,\mathrm{spores}=\text{no spores}\times \mathrm{no}\,\,\mathrm{fields}\,\mathrm{counted}\times 25\times 10^{4}$$

### Evaluation of Microparticle Photostability

The fungus *T. harzianum* (at a concentration of 1:1 v:v) and five wet and dry calcium alginate microparticles containing *T. harzianum* were exposed to ultraviolet light (UVA 400–315 nm/UVB 315–280 nm) for 24 h, at ambient temperature and at a distance of 20 cm, in a darkroom. Samples were removed at different times (0, 5, 30, 60, 120, 240, and 1440 min) and plated onto PDA (K25-610102, KASVI), prepared according to the manufacturer’s instructions.

### Release Kinetics

The spore release profile was determined using density measurements of the samples at different growth stages, starting after hydration of the microparticles (Batista et al., 2017). The microparticles were placed in PDA culture medium, where they were kept until full development of the fungus. Images were acquired at different times (0, 1, 2, 4, 8, 12, 24, and 48 h), using a photodocumentation system, and were analyzed using ImageJ software. The release was determined using Eq. 3 (Batista et al., 2017).

(3) $$\text{Release}\,\left(t\right)=\frac{A\,r\,e\,a\,\left(t\right)-\text{Area}\,\left(t_{\mathrm{microparticle}\,\mathrm{hydration}}\right)}{\text{Area}\left(t\to \infty \right)-\text{Area}\left(t_{\mathrm{microparticle}\,\mathrm{hydration}}\right)}$$

where Area(*t*) is the fungus growth area at a specific time, *t*_*microparticle hydration*_ is the time required for the hydration process to be complete, and *t*→∞ is the total area of the Petri dish.

The microparticles were exposed to UV radiation (UVA 400–315 nm/UVB 315–280 nm) at room temperature and at a distance of 20 cm. The assay was performed in triplicate.

### Enzymatic Activity

#### Chitinase Activity

The chitinase activity of the fungus *T. harzianum* encapsulated or not in the wet and dry calcium alginate microparticles was measured by detecting positive chitinase in agar (Agrawal and Kotasthane, 2012). The culture medium for the detection of chitinase contained (per liter): 0.3 g of magnesium sulfate heptahydrate (Synth), 3 g of ammonium sulfate (Synth), 2 g of monobasic potassium phosphate (Synth), 1 g of citric acid monohydrate (Dinâmica), 15 g of agar (Kasvi), 200 μL of tween 80 (Sigma-Aldrich), 4.5 g of colloidal chitin (Sigma-Aldrich) and 0.15 g of bromocresol purple (Synth). Subsequently, the pH of the solution was adjusted to 4.7 and autoclaved at 121°C for 15 min.

The culture medium was placed in Petri dishes until solidified, then wet and dry microparticles containing the encapsulated *T. harzianum* and *T. harzianum* were placed on the culture medium and incubated for 3 and 5 days at 25 ± 2°C. After this period, chitinase activity was observed by changing the color of the culture medium.

#### Cellulase Activity

The cellulase activity of the fungus *T. harzianum* and of the wet and dry microparticles containing *T. harzianum* was determined through qualitative quantification (Pansa, 2017). For this, the fungus encapsulated or not plated in culture medium selective for cellulase, using the CMC medium (0.5 g sodium nitrate (Synth), 1 g dibasic potassium phosphate (Synth), 0.5 g magnesium heptahydrate (Synth), 0.01 g iron sulfate heptahydrate (Synth), 1 g yeast extract (Kasvi), 10 g carboxymethylcellulose (Sigma-Aldrich), 15 g agar (Kasvi), 1 g H_2_O) and supplemented with 1 mL Triton X-100. Subsequently, the plates were incubated for 5 days at 30°C and a solution of red congo (0.1% v/v – Synth) added to the petri dish. After 15 min, the plates were washed with 1M NaCl solution, and cellulose degradation was observed visually as a halo.

### Biological Activity (*In vitro* Antagonism)

The biological activity of the calcium alginate microparticles containing *T. harzianum* was evaluated against the phytopathogen *S. sclerotiorum* (white mold) using an *in vitro* test. The wet or dry microparticles containing *T. harzianum* (10^8^ conidia mL^–1^) were placed on one side (right) of a Petri dish containing PDA medium, while the pathogen was placed on the opposite side (left). The plates were incubated at room temperature and the growth of the organisms was observed. The assays were performed in triplicate.

The inhibition was evaluated using a classification scale from 1 to 5 (Bell et al., 1982): class 1 = *T. harzianum* fully combated the pathogen and covered the entire surface of the medium; class 2 = *T. harzianum* occupied at least two-thirds of the surface of the medium; class 3 = *T. harzianum* and the pathogen each colonized half of the medium (or more than one-third, but less than two-thirds); class 4 = the pathogen colonized at least two-thirds of the surface of the medium and appeared to resist invasion by *T. harzianum*; class 5 = the pathogen completely overcame the *T. harzianum* and occupied the entire surface of the medium. *T. harzianum* was considered a pathogen antagonist when the mean score was ≤2, but not so antagonistic when the mean score was ≥ 3 (Bell et al., 1982).

The efficiency index for the action of *T. harzianum* against *S. sclerotiorum* was calculated using Eq. 4.

(4) $$I\,e\,f=\frac{A_{S}}{A_{T}}$$

where *A*_*S*_ is the growth area of *S. sclerotiorum* and *A*_*T*_ is the growth area of *T. harzianum*.

## Results

### Characterization of the Microparticles

**FIGURE 1:**
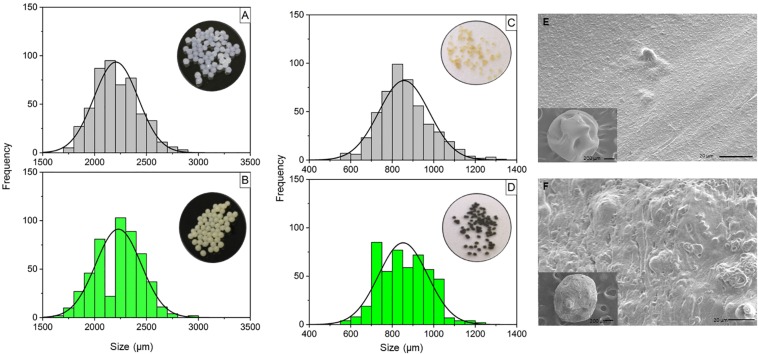
Size distributions of wet and dry calcium alginate microparticles synthesized by ionic gelation, with or without *T. harzianum* and scanning electron micrographs of the dry calcium alginate microparticles, with and without encapsulated *T. harzianum*. **(A)** Wet calcium alginate microparticles; **(B)** wet calcium alginate/fungus microparticles (1:1 w:w); **(C)** dry calcium alginate microparticles; **(D)** dry calcium alginate/fungus microparticles (1:1 w:w). The size distribution was determined using ImageJ software. The wet and dry microparticles presented mean sizes of 2000 and 800 μm, respectively. **(E)** Calcium alginate microparticles; **(F)** calcium alginate:fungus microparticles (1:1 w:w). The interiors of the microparticles with and without the fungus showed differences, due to the presence of the fungus (spores) in the second image. The images were acquired at magnifications of 95× (insert) and 250×.

The results of physicochemical characterization of the calcium alginate microparticles prepared with or without the fungus are shown in Figure 1. The calcium alginate microparticles had a spherical shape and a slightly transparent white color, while the microparticles containing the encapsulated *T. harzianum* were also spherical, and had a greenish color.

Size distribution measurements were made of the wet and dry calcium alginate microparticles prepared with or without the encapsulation of *T. harzianum*. The size distributions of the wet microparticles are shown in Figures 1A,B. The wet calcium alginate microparticles presented a size distribution from 1500 to 2700 μm, and a similar size distribution was obtained when the microparticles were prepared with the encapsulated fungus (fungus:polymer ratio of 1:1 w:w).

The size distributions of the microparticles after the drying process are shown in Figures 1C,D. The dried calcium alginate microparticles presented a size distribution from 600 to 1300 μm, while the dried microparticles containing the encapsulated *T. harzianum* showed a size distribution from 400 to 1200 μm.

#### Scanning Electron Microscopy

Scanning electron microscopy was used to characterize the morphology of the microparticles. The micrographs (Figures 1E,F) showed that the particles produced under all the synthesis conditions were spherical, even after the dehydration process. In the absence of the encapsulated fungus, the calcium alginate microparticles presented an average diameter of 905 μm, while in the presence of *T. harzianum*, encapsulated at a ratio of 1:1 (w:w), the average diameter was 812 μm. The surfaces of the microparticles presented cavities that could have been formed during the sample drying process and the preparation for microscopy analysis.

#### Differential Scanning Calorimetry

Thermograms of the microparticles with or without the encapsulated fungus, the different components, and *T. harzianum* are shown in Supplementary Figure S1, where the heat flux (W/g) is plotted as a function of temperature (°C).

The sodium alginate thermogram presented an endothermic peak at 122°C and an exothermic peak at 240°C (Supplementary Figure S1 – Alginate). The calcium chloride thermogram presented two endothermic peaks, the first at 180°C, and the second at 220°C (Supplementary Figure S1 – Calcium chloride). The thermogram of *T. harzianum* (Supplementary Figure S1 – *T. harzianum*) showed an endothermic peak at 120°C and an exothermic peak at 310°C.

The curve for the calcium alginate microparticles only showed an endothermic peak at 190°C (Supplementary Figure S1 – Microparticles), while the calcium alginate microparticles containing the encapsulated fungus presented an endothermic peak at 200°C and an exothermic peak at 300°C (Supplementary Figure S1 – Microparticles with fungus). The physical mixture of alginate and calcium chloride (Supplementary Figure S1 – Alginate and Calcium chloride) showed an endothermic peak at 163°C, while the physical mixture of sodium alginate, calcium chloride, and the fungus showed a significant endothermic peak at 190°C (Supplementary Figure S1 – Alginate, Calcium chloride and *T. harzianum*).

#### Infrared Spectroscopy

The infrared spectra of the microparticles (with or without the encapsulated fungus), the individual components, and *T. harzianum* are shown in Supplementary Figure S2. The alginate spectrum (Supplementary Figure S2 – Alginate) showed a band at 3300 cm^–1^, due to OH bonds, while bands at around 1593 and 1408 cm^–1^ could be attributed to COO^–^ bonds. The infrared spectrum of *T. harzianum* (Supplementary Figure S2 – *T. harzianum*) showed two peaks, at 3687 and 3619 cm^–1^.

The spectrum of the calcium alginate microparticles (Supplementary Figure S2 – Microparticles) showed an increased size of the band corresponding to OH bonds, due to the presence of water in the microparticles (5% moisture content). One of the bands corresponding to COO^–^ bonds was shifted to a wavelength greater than 1420 cm^–1^. Shift of the COO^–^ band confirmed the crosslinking of Ca^2+^ with the COO^–^ group of sodium alginate. When the fungus was encapsulated (Supplementary Figure S2 – Microparticles with *T. harzianum*), the presence of a single peak and a shift to 3681 cm^–1^ were indicative of interaction of the fungus with the microparticles. The spectrum for the physical mixture of sodium alginate and calcium chloride (Supplementary Figure S2 – Alginate and Calcium chloride) showed bands at 3384 and 1623 cm^–1^, which were characteristic of each substance. The physical mixture of sodium alginate, calcium chloride, and the fungus (Supplementary Figure S2 – Alginate, Calcium chloride and *T. harzianum*) showed the presence of bands corresponding to alginate (at 3300, 1593, 1408, and 1019 cm^–1^), calcium chloride and the fungus (at 3687 and 3619 cm^–1^).

### Effect of the Microparticles on Soil Microbiota

The quantification of genes associated with the nitrogen cycle was used to elucidate possible effects caused by *T. harzianum* and to determine whether the encapsulation of this fungus could also lead to alteration of the original microbiota of the soil.

**FIGURE 2:**
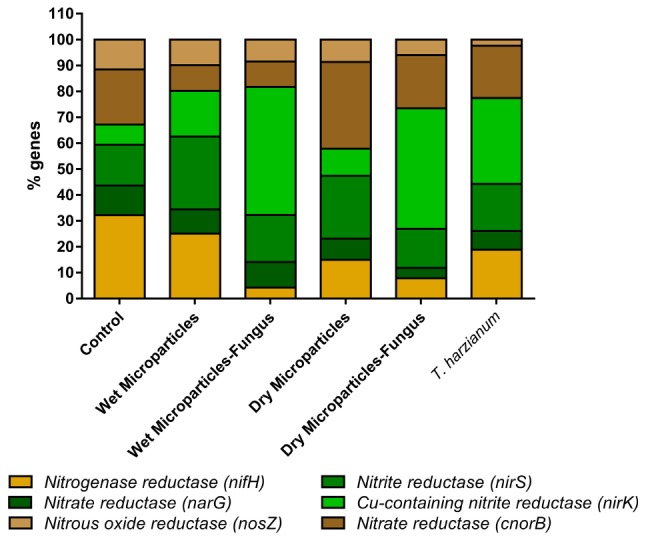
Molecular evaluation of the soil microbiota, in terms of the proportions of genes obtained using real-time PCR, after 30 days of treatment. The results showed that there was a decrease of the nitrogen-fixing bacteria and increase of the denitrifiers, compared to the negative control, while there was no change in the soil microbiota, comparing the encapsulated and unencapsulated *T. harzianum.*

The results of the molecular evaluation of the soil microbiota using real-time PCR are shown in Figure 2.

In terms of the proportions of genes linked to the nitrogen cycle (Figure 2), a decrease of nitrogen-fixing bacteria were observed for the treatments, compared to the negative control. At the same time, the proportions of denitrification bacteria increased for all the treatments, compared to the negative control. However, comparison of the results for the encapsulated and unencapsulated fungus revealed no change in the proportions of the genes involved in the nitrogen cycle.

### Quantification of Spores

**FIGURE 3:**
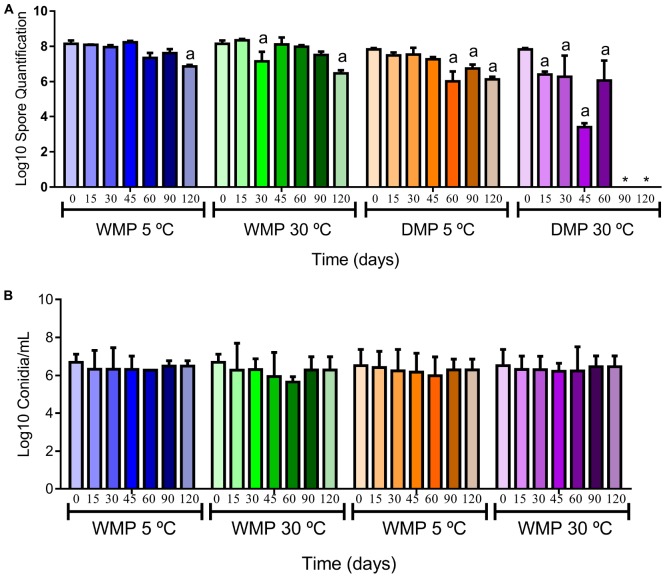
Quantification of the spores of *T. harzianum* encapsulated in the wet (WMP) and dry (DMP) calcium alginate microparticles, during storage for 90 days at 5 and 30°C, using the CFU g^–1^ and Neubauer chamber techniques. **(A)** Log10 spore quantification using the CFU g^–1^ method applied to the wet and dry microparticles; **(B)** Log10 quantification of spores (conidia/mL) using the Neubauer chamber method applied to the wet and dry microparticles. The encapsulated fungus presented higher spore viability during the period evaluated. * No conidia growth at this time. ^a^ refers to the significant difference in the quantification of spores on the evaluated days when compared to day 0.

Figure 3 shows the results of spore quantification for the wet and dry calcium alginate microparticles containing *T. harzianum*, following storage at different temperatures (5 and 30°C), using the CFU g^–1^ and Neubauer chamber counting methods.

The microparticles containing the encapsulated *T. harzianum* fungus showed viability of 10^8^ and 10^7^ CFU mL^–1^ for wet and dry microparticles, respectively. When CFU g^–1^ was used (Figure 3A), wet microparticles stored at 5°C did not show significant variation in spore viability at 15, 30, 45, 60 and 90 days. However, after 120 days of storage, a significant variation of the spores (10^6^ CFU mL^–1^) was observed. Compared to this, when the wet microparticles were stored at 30°C, there was a significant variation in the number of spores after 30 days (10^7^ CFU mL^–1^). The dried microparticles stored at 5°C also showed significant variation in spore viability after 60 days of storage (10^6^ CFU mL^–1^). When the dried microparticles were stored at 30°C, they showed a decrease in the spore numbers after 15 days. After 90 days, the spores were no more viable.

In order to evaluate the spore viability by another methodology, we used the Neubauer chamber (Figure 3B). Initially the microparticles containing the encapsulated fungus *T. harzianum* presented viability of 10^6^ CFU mL^–1^ for wet and dry microparticles. The results obtained by counting in a Neubauer chamber showed that the wet and dry microparticles stored at 5 and 30°C maintained spore viability after 120 days of storage (10^6^ CFU mL^–1^). The encapsulated spores presented better viability in the wet microparticles at both stored temperatures. The dry microparticles presented better viability of the stored spores at 5°C.

### Evaluation of Photostability

**FIGURE 4:**
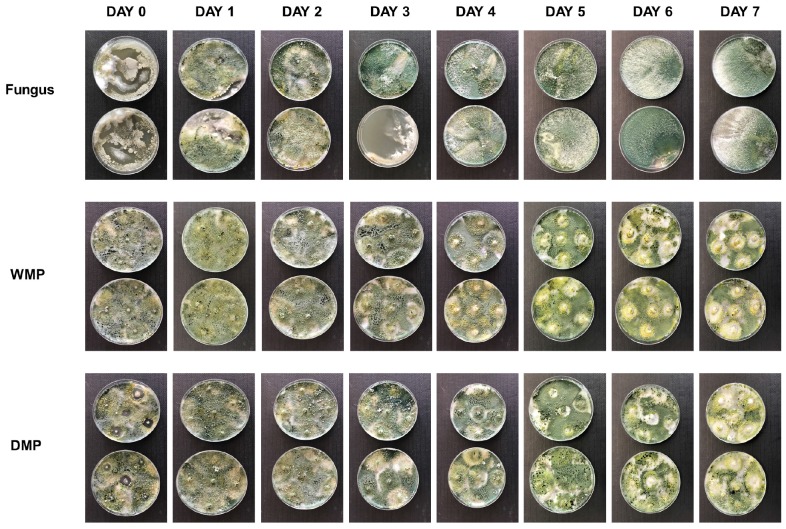
Photostability assays after 7 days exposure to ultraviolet radiation, for the free fungus and the wet (WMP) and dry (DMP) microparticles containing encapsulated *T. harzianum*. The results showed that encapsulation of the fungus provided protection against ultraviolet radiation.

The results (Figure 4) showed that growth of the free fungus was negatively affected after 3 days of exposure, compared to growth of the organism encapsulated in the wet or dry microparticles.

After encapsulation, the fungus *T. harzianum* also showed a better resistance to ultraviolet radiation when compared to non-encapsulated fungus.

### Release Kinetics

**FIGURE 5:**
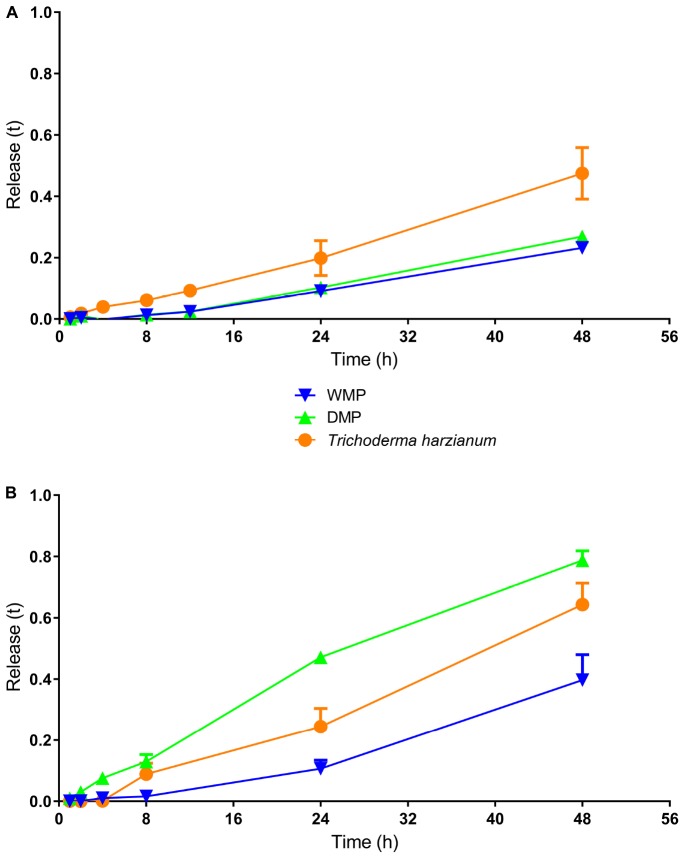
The release kinetics assays using the wet and dry microparticles with encapsulated *T. harzianum*, as well as the unencapsulated fungus, without **(A)** or with **(B)** exposure to ultraviolet radiation. The encapsulated fungus presented slower release.

It was also important to evaluate the releases kinetics of the microcapsule contents. For this, a template was used as described by Batista et al. (2017). The results of the growth kinetics assays for the fungus encapsulated in the wet and dry calcium alginate microparticles, as well as the unencapsulated fungus, are shown in Figure 5. In the case of the dry microparticles, the measurements were only considered after the swelling process was complete. A larger growth area was observed for the unencapsulated *T. harzianum*, compared to the fungus encapsulated in the microparticles (wet or dry), demonstrating the sustained release of the fungus contained in the calcium alginate microparticles. After exposure of the microparticles and the fungus to ultraviolet radiation, a smaller area of growth was shown by the unencapsulated fungus, compared to the fungus encapsulated in dry microparticles (Figure 5B). This confirmed the protection provided to the fungus by the microparticles, with the growth of the organism not being affected by the ultraviolet radiation treatment.

### Enzymatic Activity

#### Chitinase Activity

The colloidal chitin culture medium containing bromocresol purple, when inoculated with *T. harzianum*, results in the breakdown of chitin into N-acetyl glucosamine. This break causes a color change in the culture medium based on the pH change, going from yellow to purple in the presence of chitinolytic activity (Agrawal and Kotasthane, 2012).

**FIGURE 6:**
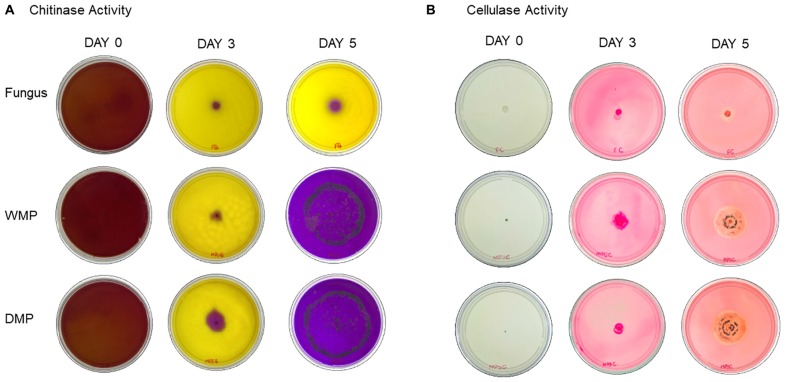
Results of **(A)** chitinase activity by detection of chitinase positive agar and **(B)** cellulase activity for *T. harzianum* fungus encapsulated or not in wet or dry calcium alginate microparticles evaluated for 3 and 5 days of incubation.

The chitinase activity was evaluated by the purple color diameter after 3 and 5 days of incubation in the fungus *T. harzianum* encapsulated or not in wet and dry calcium alginate microparticles (Figure 6A). *T. harzianum* showed an increase in chitinase activity after microencapsulation and this can be observed after 3 and 5 days of incubation, evidencing a higher activity after 5 days.

After 3 days of incubation, the wet and dry microparticles containing the fungus *T. harzianum* showed a higher chitinolytic activity than the non-encapsulated fungus, noting that the dry microparticles also showed higher activity in this period. However, after 5 days of incubation, we noticed that there was a total color change in the culture medium where the wet and dry microparticles containing the encapsulated fungus were present with no difference between them.

#### Cellulase Activity

After incubation of the plates in CMC medium, the plates were stained with red congo and thus the degradation halo was revealed. The degradation halo was formed by the absence of dye binding with β-1,4-glycidyl bonds (Florencio, 2011), indicating the areas of hydrolysis. This method is used as a simple tool for the identification of microorganisms producing cellulase enzymes (Ten et al., 2004).

The results of the evaluation of cellulase activity by qualitative quantification can be seen in Figure 6B.

It can be seen that there was formation of the halo hydrolysis in the plates containing *T. harzianum* encapsulated or not in wet and dry microparticles. When the fungus was microencapsulated, there was an increase in the colony and, consequently, in the halo of degradation when compared to the non-encapsulated fungus.

### Biological Activity (*In vitro* Antagonism)

**FIGURE 7:**
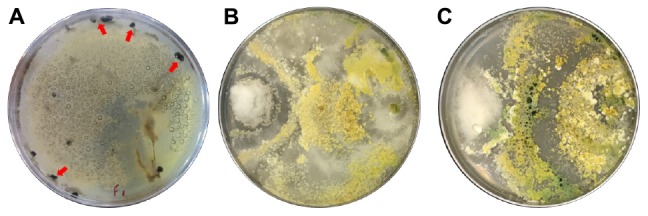
The *in vitro* antagonism assays using *T. harzianum* against the phytopathogenic fungus *S. sclerotiorum* (white mold). **(A)** Results obtained with the unencapsulated fungus, showing that the fungus was able to control the phytopathogen, according to the classification scale (Bell et al., 1982), but that there was the formation of white mold sclerotia (indicated by the arrows). **(B)** Results using the fungus encapsulated in wet microparticles. **(C)** Results using the fungus encapsulated in dried microparticles, indicating that the encapsulated fungus was a satisfactory antagonist.

The *in vitro* assays were performed using unencapsulated *T. harzianum* and with the fungus encapsulated in the calcium alginate microparticles. The results are illustrated in Figure 7.

The unencapsulated *T. harzianum* showed class ≥3 on the antagonism classification scale, indicating that the fungus was moderately antagonistic in this assay, to the classification of Bell et al. (1982). Although the unencapsulated fungus was antagonistic to the phytopathogen, there was sclerotia formation by *S. sclerotiorum* in this sample (Figure 7A).

The fungus encapsulated in the wet and dry calcium alginate microparticles was able to control the growth of *S. sclerotiorum* in the *in vitro* trials. The classification was ≤ 2 and there was no formation of sclerotia (Figures 7B,C).

The results showed that there was a difference in the classification scale of the encapsulated and unencapsulated *T. harzianum* fungus. This difference is possibly due to the fact that microencapsulation provides protection of biotic and abiotic factors, as well as increased efficacy as a biological control agent.

The efficiency index (Supplementary Table S2) showed that the fungus encapsulated in the wet microparticles had 20-fold higher efficiency in the control of *S. sclerotiorum*, compared to the unencapsulated fungus, with no sclerotia formation. These results indicate that the encapsulated fungus is more promising for the control of *S. sclerotiorum*, compared to the unencapsulated fungus.

## Discussion

In this study, the synthesis and characterization of calcium alginate microparticles through the ionic gelation method containing spores of the fungus *T. harzianum* was performed. The calcium alginate microparticles synthesized by the ionic gelation method obtained an average size of 900 μm, spherical morphology and were able to encapsulate the *T. harzianum* fungus. Microparticles composed of sodium alginate, sodium polyphosphate and glycerol, in different proportions were used to encapsulated containing *T. harzianum.* They were encapsulated by ionic gelation methodology as the same used in this study (dos Santos et al., 2015). The FTIR characterization showed a peak at around 1019 cm^–1^ is characteristic of C-O-C bonds. Locatelli et al. (2018) also found these characteristic bands of sodium alginate. Bands associated with the presence of calcium chloride (Supplementary Figure S2 – Calcium chloride) appeared at 3428 and 1617 cm^–1^, which relate to the vibration of OH functional group (Jurić et al., 2019).

Subsequently, the molecular analysis of the soil microbiota was carried out with bacterial genes involved in the stages of the nitrogen cycle. Thus, each part of the nitrogen cycle has a group of responsible bacteria. These bacteria are of great importance in agriculture, since they are responsible in transforming the nitrogen in forming bioavailable, thus making the soil fertile (Hirsch and Mauchline, 2015). However, little is know about the effects of *T. harzianum* encapsulated in nitrogen cycle microorganisms as well as the effects of microparticles.

In this study, the soil microbiota had an increase in denitrifying bacteria from the nitrogen cycle in the presence of the microparticles containing the encapsulated *T. harzianum* fungus when we compared with the negative control. As example, Masrahi et al. (2014) evaluated the influence of silver nanoparticles on the kinetics of nitrification in the soil, using the soil-mud nitrification method in combination with sorption and dissolution isotherms. It was found that Ag^+^ and silver nanoparticles (AgNPs) acted to suppress the nitrification process, with the degree of suppression increasing as the nanoparticle concentration increased. However, the mechanism by which the AgNPs exerted toxicity toward the nitrifying bacteria remained unclear. In other work, Maurer-Jones et al. (2013) evaluated the toxicity of quantum dots toward the expression of bacterial genes involved in the nitrogen cycle. The presence of the quantum dots altered the expression of these genes, and also modified the processes of nitrification/denitrification. Priester et al. (2012) investigated the susceptibility of soybean to cerium oxide nanoparticles (nano-CeO_2_). At high concentrations, the nano-CeO_2_ decreased N_2_ fixation by 80%, compared to the negative control, while at low nano-CeO_2_ concentrations, the N_2_ fixation potential appeared to increase.

In another study, Guilger et al. (2017) evaluated the effect on the soil microbiota of biogenic silver nanoparticles produced using *T. harzianum*. The authors reported that the amount of bacteria increased over time in all soil samples tested, as compared to the negative control. After 30 days of exposure, the soil shown largest difference of the number of bacteria. However, after 90 days, there was a decreasing number of fixing and denitrifying bacteria.

These studies have shown that the presence of microparticles in the soil increases the amount of denitrifying bacteria in the soil. This means that the amount of nitrogen returned to the atmosphere is higher and thus, it becomes available for nitrogen fixation. In addition, the genus *Trichoderma* is able to reduce the amounts of nitrogen fertilizers in agriculture and helps in the growth of plants (Harman, 2011). This stage of toxicity assessment is extremely important for the development of new products that will one day be available in the market. Therefore, the use of the microparticles containing the encapsulated fungus in agriculture turns out to be promising.

Another important aspect is the viability of the *T. harzianum* spores in the microparticles. Here was evaluated in this work, where it had a viability of 120 days in two different storage temperatures (5 and 30°C). The encapsulated spores presented better viability in the wet microparticles at both stored temperatures. The dry microparticles presented better viability of the stored spores at 5°C. As described by Locatelli et al. (2018) and dos Santos et al. (2015), our viability results are in line, since it was possible to maintain the viability of encapsulated *T. harzianum* spores.

Another important point that interferes with the effect of the *T. harzianum* biocontrol agent is UV radiation. For this, a photostability test was carried out and the microparticles were able to evaluate the fungus protection. *T. harzianum* is a well-recognized biological control agent, but there are limitations associated with its use that can prejudice its effectiveness, such as sensitivity to UV light and unsuitable humidity and/or temperature. These factors can lead to low persistence of the organism in the environment (Vemmer and Patel, 2013). Microencapsulation provides the biological control agent with protection against biotic and abiotic factors, including UV light, temperature, and humidity. In addition, the slow and controlled release results in increased persistence of the agent in the environment (Paulo and Santos, 2017).

After encapsulation, the fungus *T. harzianum* showed a better response to ultraviolet radiation when compared to non-encapsulated fungus through exposure, where it is sensitive. In the present case, it could be seen that the amount of fungal growth on the plate was lower for the unencapsulated organism, compared to the growth of the encapsulated fungus, especially after 5 days of exposure to UV radiation. UV radiation is one of the main environmental factors causing direct or indirect damage to the fungi, reducing their effectiveness and inhibiting both germination of conidia and the early stages of germ tube development (Braga et al., 2002; Hughes et al., 2003; Ozeçelik, 2007). Considering the growth of the fungus exposed to UV radiation for the period employed here (7 days), it is evident that encapsulation of *T. harzianum* in calcium alginate microparticles provides protection against UV radiation.

Elçin (1995) encapsulated *Bacillus sphaericus* 2362 in calcium alginate microcapsules, in order to evaluate their photoprotective effect. The microcapsules containing the encapsulated bacteria, as well as a suspension of these microcapsules, were exposed for 48 h to UV radiation from a 12 W lamp, at ambient temperature. It was found that the viability of the unencapsulated spores decreased from 1 × 10^8^ to 1 × 10^3^ CFU mL^–1^ after 12 h of exposure. For times over 24 h, the encapsulated spores presented greater viability, compared to the free spores. At 1% (m:v) in the alginate microcapsules, the number of viable spores was 10 times greater, compared to the free spores. The results demonstrated that encapsulation increased the viability of the spores and made them less sensitive to ultraviolet radiation, confirming the photoprotective effectiveness of the system.

Hughes et al. (2003) investigated the effect of solar radiation (UV-B, UV-A – 315 to 400 nm and photosynthetically radiation – 400 to 700 nm) on five different fungi and oomycetes (*Geomyces pannorum*, *Phoma herbarum*, *Pythium* sp., *Verticillium* sp., and *Mortierella parvispora*). It was found that after 3 h of exposure to solar radiation (> 287 nm), hyphal extension rates decreased by 100% (*M. parvispora*), 63% (*Verticillium* sp.), 48% (*G. pannorum*), 41% (*Pythium* sp.), and 15% (*P. herbarum*), compared to controls that did not receive radiation. Therefore, the exposure to all types of UV radiation was detrimental to fungal growth.

Ozeçelik (2007) evaluated the effect of UV radiation (254 and 354 nm) on the growth of different fungi and bacteria on various types of surfaces. The organisms became inactive after 45 min of exposure at 254 nm, while exposure at 354 nm did not affect the growth of the fungi and bacteria.

Comparison of the results obtained for the wet and dry microparticles containing the encapsulated *T. harzianum* showed that there were no differences between them, with both types of microparticle providing protection against UV radiation. And from the commercial perspective, the dried microparticles containing the encapsulated fungus would be more practical, due to their greater ease of storage and application.

These results were able to show us that the system of this work is quite effective, because it presents a good stability and viability of the spores even in the presence of UV radiation.

After encapsulation, it is important to evaluate the release kinetics of the compound and for this a template was used as described by Batista et al. (2017). The release kinetics assay showed us how the fungus release from the calcium alginate microparticles in the presence or absence of ultraviolet light. The fungus in absence of UV presented a larger release area than the encapsulated fungus, this shows the controlled release of the microparticles. The fungus had a larger release area when encapsulated and exposed to UV, this happened due to the fact that the microparticle system used in this work is effective in protection, especially in dry microparicles. Batista et al. (2017) evaluated the growth areas of the *Beauveria bassiana* fungus encapsulated in alginate-bentonite microparticles dried using different air flow and lyophilization processes. The results showed that the light emission was directly related to the development of the organism and that the growth area was larger when the microparticles were dried by the air flow method. Elsewhere, He et al. (2015) encapsulated the bacteria *Raoultella panticola* Rs-2 in alginate-bentonite microparticles and found that the organism presented behavior that was dependent on the concentrations of alginate and bentonite.

As a consequence, we can show that the fungus encapsulated in microparticles presents a great potential for protection against UV radiation and this is a very important factor for the use of these microparticles under field conditions. Once this was understood, a biological activity assay was performed to evaluate whether the fungus, even after microencapsulation, remained a good biological control agent.

*Trichoderma harzianum* is one of the most widely used biological control agents in agriculture as it has potential against a wide range of fungal pathogens. In this study, we investigated the antagonistic activity of *T. harzianum* and microparticles containing *T. harzianum* encapsulated against *S. sclerotiorum*. The *in vitro* antagonism assay showed an improvement in the efficacy of action against the phytopathogen *S. sclerotiorum* when encapsulated, where there was inhibition of phytopathogen growth and absence of sclerotia. Thus, we can observe that *T. harzianum* encapsulated presented a significant improvement in the biocontrol potential. Based on this comments, the results showed us that the encapsulated fungus had a greater antagonistic activity against wild mold than the unencapsulated one. Therefore, this does not mean that *T. harzianum* showed no activity on the pathogen, but that encapsulation provided an improvement in its action. Zhang et al. (2016) evaluated the biocontrol potential of the *T. harzianum* isolate T-aloe against the phytopathogen *S. sclerotiorum*. The results showed that the *T. harzianum* inhibited the growth of *S. sclerotiorum* with na efficiency of 56.3% in dual culture tests. In plate tests, there was na inhibition of 51.2% and there was production of sclerotia.

The enzymatic activity is a key point in the mechanism of action of *T. harzianum* (Druzhinina et al., 2011), and our results have shown that the encapsulation promotes an increase in the chitinase and cellulase activities of the encapsulated *T. harzianum*. This could be due the fact that the fungi are protected in the microparticles which makes then less exposed to external factors (UV, temperature, pH, etc.). Agrawal and Kotasthane (2012) evaluated the chitinolytic activity of different isolates of *Trichoderma* from different geographical locations in Central India. Their results showed that 61 isolates showed chitinolytic activity and were classified into 4 different groups (no chitinase activity, low chitinase activity, medium chitinase activity and high chitinase activity). Of the 61 isolates, 17 presented high chitinase activity and 8 presented medium chitinase activity. Ting and Chai (2015) evaluated the expression of chitinase and β-1,3-glucanase activity of *T. harzianum* in the presence of pathogenic isolates (*Fusarium oxysporum*, *Ganoderma boninense*) and non-patogenics (*T. viridescens*, *Serratia marcescens*, and *Streptomyces griseus*). The results showed that *T. harzianum* presented chitinase activity, independent of the presence of pathogen isolates, as it produces these enzymes naturally.

*In vitro* assays are essential for the evaluation of results, as they are reliable results and minimize possible impacts that *in vivo* tests may cause. Thus, the literature has shown that there are no differences when comparing the results for *in vivo* and *in vitro* assays. Moya et al. (2020) evaluated native species of *Trichoderma* spp. as biological control agents for *Pyrenophora teres* through *in vitro* and *in vivo* assays, where *in vitro* assays were done through antagonism and *in vivo* assays were done in greenhouses. Results from *in vitro* assays showed that all *Trichoderma* isolates were able to inhibit mycelial pathogen growth by up to 54% when compared to the control. Meanwhile, *in vivo* results also showed that all *Trichoderma* isolates were able to significantly decrease the pathogen incidence in trough seedlings by up to 55%. Thus, this study shows us that there is no difference in results between *in vitro* and *in vivo* assays. In addition, the results showed that the same isolates that showed better antagonism in the *in vitro* assay were also the best *in vivo*. Therefore, *in vitro* assays are needed to evaluate the biological mechanisms involved in antagonism and to confirm this mechanism in *in vivo* assays.

The tests were performed with two types of microparticles containing the encapsulated fungus (wet and dry). We can highlight that there is a difference in the results between them. Comparisons of the main results can be seen in Table 2.

**TABLE 2:** Comparison of the main results of the tests performed with wet and dry microparticles. The (=) symbol demonstrates that there is no difference in results between the microparticles and the (+) symbol demonstrates that one of the microparticles was better than the other.

	**Shelf life**	**Effect on soil microbiota**	**Photostability**	**Release kinetics**	**Enzymatic activity**	**Biological activity**
Wet microparticles	+	=	=	=	=	=
Dry microparticles		=	=	=	=	=

Overall, there are no differences between wet and dry microparticles when comparing the results of biological activity, photostability, among others. However, when we evaluate shelf life results we see that wet microparticles have a better shelf life when compared to dry microparticles. Thus, from the point of view of stability it would be better to use wet microparticles.

## Conclusion

In summary, the results of this study have shown that calcium alginate microparticles are effective in protecting *T. harzianum* against ultraviolet radiation and potentially other abiotic factors in the environment. The results of DSC and FTIR analyses revealed that the fungus interacted with the calcium alginate microparticles. Molecular evaluation of the soil microbiota showed that the microparticles increased the number of denitrifying bacteria and *T. harzianum* with the potential to decrease the use of nitrogen fertilizers. In release experiments, the encapsulated fungus presented sustained release. When exposed to ultraviolet radiation, the unencapsulated fungus showed a smaller area of growth compared to the fungus encapsulated in dry microparticles. This further confirmed the protection provided to the fungus by the microparticles, against ultraviolet radiation. The *in vitro* antagonism assays showed that the wet microparticles containing the encapsulated fungus provided greater control of the white mold phytopathogen, compared to the unencapsulated fungus, with no formation of sclerotia. The encapsulation of the fungus *T. harzianum* also showed an improvement in the chitinolytic and cellulosic activity, and therefore a potential for use in agriculture. Overall, the results point out to the possibility for the use of formulations comprising encapsulated fungus for use in agricultural applications.

## Data Availability Statement

The datasets generated for this study are available on request to the corresponding author.

## Author Contributions

CM contributed to the design of the experiments, trial implementation, data collection, data processing, and manuscript writing. NB-J contributed to the performance of the experiments and data collection. RL and LF supervised and guided all the steps for the development of the manuscript, contributed to the design of the experiments, treatment and interpretation of data, and manuscript writing.

## Conflict of Interest

The authors declare that the research was conducted in the absence of any commercial or financial relationships that could be construed as a potential conflict of interest.

## References

[B1] Adzmi F. Meon S. Musa M. H. Yusuf N. A. (2012). Preparation, characterisation and viability of encapsulated *Trichoderma harzianum* UPM40 in alginate-montmorillonite clay. *J. Microencapsul.* 29 205–210. 10.3109/02652048.2012.659286 22309479

[B2] Agrawal T. Kotasthane A. S. (2012). Chitinolytic assay of indigenous *Trichoderma* isolates collected from different geographical locations of Chhattisgarh in Central India. *SpringerPlus* 1:73. 10.1186/2193-1801-1-73 23526575 PMC3602610

[B3] Batista D. P. C. de Oliveira I. N. Ribeiro A. R. B. Fonseca E. J. S. Santos-Magalhães N. S. de Sena-Filho J. G. (2017). Encapsulation and release of *Beauveria bassiana* from alginate–bentonite nanocomposite. *RSC Adv.* 7 26468–26477. 10.1039/C7RA02185B

[B4] Bell D. K. Wells H. D. Markham C. R. (1982). In vitro antagonism of *Trichoderma* species against six fungal plant pathogens. *Phytopathology* 72 379–382.

[B5] Błaszczyk L. Siwulski M. Sobieralski K. Lisiecka J. Jędryczka M. (2014). *Trichoderma* spp. – application and prospects for use in organic farming and industry. *J. Plant Prot. Res.* 54 309–317. 10.2478/jppr-2014-0047

[B6] Braga G. U. L. Rangel D. E. N. Flint S. D. Miller C. D. Anderson A. J. Roberts D. W. (2002). Damage and recovery from UV-B exposure in conidia of the entomopathogens *Verticillium lecanii* and Aphanocladium album. *Mycologia* 94 912–920. 10.1080/15572536.2003.11833149 21156565

[B7] Carvalho D. D. C. Geraldine A. M. Junior M. L. de Mello S. C. M. (2015). Controle biológico do mofo-branco por *Trichoderma harzianum* em feijão em condições de campo. *Pesqui. Agropecuária Bras.* 50 1220–1224.

[B8] dos Santos G. F. Locatelli G. O. Coêlho D. A. Botelho P. S. Amorim M. S. de (2015). Factorial design, preparation and characterization of new beads formed from alginate, polyphosphate and glycerol gelling solution for microorganism microencapsulation. *J. Solgel Sci. Technol.* 75 345–352. 10.1007/s10971-015-3705-5

[B9] Druzhinina I. S. Seidl-Seiboth V. Herrera-Estrella A. Horwitz B. A. Kenerley C. M. Monte E. (2011). *Trichoderma*: the genomics of opportunistic success. *Nat. Rev. Microbiol.* 9 749–759. 10.1038/nrmicro2637 21921934

[B10] Elad Y. (2000). Biological control of foliar pathogens by means of *Trichoderma harzianum* and potential modes of action. *Crop Prot.* 19 709–714. 10.1016/s0261-2194(00)00094-6

[B11] Elçin Y. M. (1995). Bacillus sphaericus 2362-calcium alginate microcapsules for mosquito control. *Enzyme Microb. Technol.* 17 587–591. 10.1016/0141-0229(94)00026-N

[B12] Florencio C. (2011). *Microrganismos Produtores de Celulases: Seleção de Isolados de Trichoderma spp. Dissertação – Programa de Pós-graduação em Biotecnologia.* São Carlos: Universidade de São Carlos.

[B13] Fraceto L. F. Maruyama C. R. Guilger M. Mishra S. Keswani C. Singh H. B. (2018). *Trichoderma harzianum*-based novel formulations: potential applications for management of Next-Gen agricultural challenges. *J. Chem. Technol. Biotechnol.* 93 2056–2063. 10.1002/jctb.5613

[B14] Guilger M. Pasquoto-Stigliani T. Bilesky-Jose N. Grillo R. Abhilash P. C. Fraceto L. F. (2017). Biogenic silver nanoparticles based on *Trichoderma harzianum*: synthesis, characterization, toxicity evaluation and biological activity. *Sci. Rep.* 7:sre44421. 10.1038/srep44421 28300141 PMC5353535

[B15] Harman G. E. (2011). Multifunctional fungal plant symbionts: new tools to enhance plant growth and productivity. *New Phytol.* 189 647–649. 10.1111/j.1469-8137.2010.03614.x 21223281

[B16] He Y. Wu Z. Tu L. Han Y. Zhang G. Li C. (2015). Encapsulation and characterization of slow-release microbial fertilizer from the composites of bentonite and alginate. *Appl. Clay Sci.* 10 68–75. 10.1016/j.clay.2015.02.001

[B17] Hermosa R. Viterbo A. Chet I. Monte E. (2012). Plant-beneficial effects of *Trichoderma* and of its genes. *Microbiology* 158 17–25. 10.1099/mic.0.052274-0 21998166

[B18] Hirsch P. R. Mauchline T. H. (2015). “Chapter two - the importance of the microbial N cycle in soil for crop plant nutrition,” in *Advances in Applied Microbiology*, eds Sariaslani S. Gadd G. M., (Cambridge, MA: Academic Press), 45–71. 10.1016/bs.aambs.2015.09.001 26505688

[B19] Hjelmsø M. H. Hansen L. H. Baelum J. Feld L. Holben W. E. Jacobsen C. S. (2014). High-resolution melt analysis for rapid comparison of bacterial community compositions. *Appl. Environ. Microbiol.* 80 3568–3575. 10.1128/aem.03923-13 24610853 PMC4054126

[B20] Howell C. R. (2003). Mechanisms employed by *Trichoderma* species in the biological control of plant diseases: the history and evolution of current concepts. *Plant Dis.* 87 4–10. 10.1094/pdis.2003.87.1.4 30812698

[B21] Huang W. Wang Y. Ren L. Du C. Shi X. (2009). A novel PHBV/HA microsphere releasing system loaded with alendronate. *Mater. Sci. Eng. C* 29 2221–2225. 10.1016/j.msec.2009.05.015

[B22] Hughes K. A. Lawley B. Newsham K. K. (2003). Solar UV-B radiation inhibits the growth of antarctic terrestrial fungi. *Appl. Environ. Microbiol.* 69 1488–1491. 10.1128/AEM.69.3.1488-1491.2003 12620833 PMC150076

[B23] Ihrmark K. Asmail N. Ubhayasekera W. Melin P. Stenlid J. Karlsson M. (2010). Comparative molecular evolution of *Trichoderma* chitinases in response to mycoparasitic interactions. *Evol. Bioinforma. Online* 6 1–26. 20454524 10.4137/ebo.s4198 PMC2865166

[B24] Jung J. Yeom J. Han J. Kim J. Park W. (2012). Seasonal changes in nitrogen-cycle gene abundances and in bacterial communities in acidic forest soils. *J. Microbiol.* 50 365–373. 10.1007/s12275-012-1465-2 22752898

[B25] Jung J. Yeom J. Kim J. Han J. Lim H. S. Park H. (2011). Change in gene abundance in the nitrogen biogeochemical cycle with temperature and nitrogen addition in Antarctic soils. *Res. Microbiol.* 162 1018–1026. 10.1016/j.resmic.2011.07.007 21839168

[B26] Jurić S. Ćermić E. Topolovec-Pintarić S. Bedek M. Vinceković M. (2019). Physicochemical properties and release characteristics of calcium alginate microspheres loaded with *Trichoderma* viride spores. *J. Integr. Agric.* 18 2–16.

[B27] Keswani C. Mishra S. Sarma B. K. Singh S. P. Singh H. B. (2014). Unraveling the efficient applications of secondary metabolites of various *Trichoderma* spp. *Appl. Microbiol. Biotechnol.* 98 533–544. 10.1007/s00253-013-5344-5 24276619

[B28] Knudsen G. R. (1990). Effects of temperature, soil moisture, and wheat bran on growth of *Trichoderma harzianum* from alginate pellets. *Phytopathology* 80:724 10.1094/phyto-80-724

[B29] Lewis J. A. Papavizas G. C. (1985). Characteristics of alginate pellets formulated with *Trichoderma* and *Gliocladium* and their effect on the proliferation of the fungi in soil. *Plant Pathol.* 34 571–577. 10.1111/j.1365-3059.1985.tb01409.x

[B30] Li Q. Ning P. Zheng L. Huang J. Li G. Hsiang T. (2012). Effects of volatile substances of *Streptomyces globisporus* JK-1 on control of *Botrytis cinerea* on tomato fruit. *Biol. Control* 61 113–120. 10.1016/j.biocontrol.2011.10.014

[B31] Locatelli G. O. dos Santos G. F. Botelho P. S. Finkler C. L. L. Bueno L. A. (2018). Development of *Trichoderma* sp. formulations in encapsulated granules (CG) and evaluation of conidia shelf-life. *Biol. Control* 117 21–29. 10.1016/j.biocontrol.2017.08.020

[B32] Mancera-López M. E. Izquierdo-Estévez W. F. Escalante-Sánchez A. Ibarra J. E. Barrera-Cortés J. (2019). Encapsulation of *Trichoderma harzianum* conidia as a method of conidia preservation at room temperature and propagation in submerged culture. *Biocontrol. Sci. Technol.* 29 107–130. 10.1080/09583157.2018.1535053

[B33] Masrahi A. VandeVoort A. R. Arai Y. (2014). Effects of silver nanoparticle on soil-nitrification processes. *Arch. Environ. Contam. Toxicol.* 66 504–513. 10.1007/s00244-013-9994-1 24487627

[B34] Maurer-Jones M. A. Gunsolus I. L. Murphy C. J. Haynes C. L. (2013). Toxicity of engineered nanoparticles in the environment. *Anal. Chem.* 85 3036–3049. 10.1021/ac303636s 23427995 PMC4104669

[B35] Moya P. Barrera V. Cipollone J. Bedoya C. Kohan L. Toledo A. (2020). New isolates of *Trichoderma* spp. as biocontrol and plant growth–promoting agents in the pathosystem *Pyrenophora* teres-barley in Argentina. *Biol. Control* 141:104152 10.1016/j.biocontrol.2019.104152

[B36] Muñoz-Celaya A. L. Ortiz-García M. Vernon-Carter E. J. Jauregui-Rincón J. Galindo E. Serrano-Carreón L. (2012). Spray-drying microencapsulation of *Trichoderma harzianum* conidias in carbohydrate polymers matrices. *Carbohydr. Polym.* 88 1141–1148. 10.1016/j.carbpol.2011.12.030

[B37] Ozeçelik B. (2007). Fungi/bactericidal and static effects of ultraviolet light in 254 and 354 nm wavelengths. *Res. J. Microbiol.* 2 42–49. 10.3923/jm.2007.42.49

[B38] Pansa C. C. (2017). *Trichoderma spp. de Solos da Floresta Amazônica Como Fonte de Enzimas Celulolíticas.* São Paulo: Universidade de São Paulo.

[B39] Paula A. R. Carolino A. T. Paula C. O. Samuels R. I. (2011). The combination of the entomopathogenic fungus *Metarhizium anisopliae* with the insecticide imidacloprid increases virulence against the dengue vector *Aedes aegypti* (Diptera: Culicidae). *Parasit. Vectors* 4:8. 10.1186/1756-3305-4-8 21266078 PMC3037915

[B40] Paulo F. Santos L. (2017). Design of experiments for microencapsulation applications: a review. *Mater. Sci. Eng. C* 77 1327–1340. 10.1016/j.msec.2017.03.219 28532010

[B41] Pazini W. C. Galli J. C. (2011). Reduction of insecticides applications through the adoption of integrated management tactics of *Triozoida limbata* (Enderlein, 1918) (Hemiptera: triozidae) in guava tree. *Rev. Bras. Frutic.* 33 66–72. 10.1590/S0100-29452011000100010

[B42] Poletto F. S. Fiel L. A. Donida B. Ré M. I. Guterres S. S. Pohlmann A. R. (2008a). Controlling the size of poly(hydroxybutyrate-co-hydroxyvalerate) nanoparticles prepared by emulsification–diffusion technique using ethanol as surface agent. *Colloids Surf. Physicochem. Eng. Asp.* 324 105–112. 10.1016/j.colsurfa.2008.04.003

[B43] Poletto F. S. Jäger E. Cruz L. Pohlmann A. R. Guterres S. S. (2008b). The effect of polymeric wall on the permeability of drug-loaded nanocapsules. *Mater. Sci. Eng. C* 28 472–478. 10.1016/j.msec.2007.04.015

[B44] Priester J. H. Ge Y. Mielke R. E. Horst A. M. Moritz S. C. Espinosa K. (2012). Soybean susceptibility to manufactured nanomaterials with evidence for food quality and soil fertility interruption. *Proc. Natl. Acad. Sci. U.S.A.* 109 E2451–E2456. 10.1073/pnas.1205431109 22908279 PMC3443164

[B45] Rodrigues S. da Costa A. M. R. Grenha A. (2012). Chitosan/carrageenan nanoparticles: effect of cross-linking with tripolyphosphate and charge ratios. *Carbohydr. Polym.* 89 282–289. 10.1016/j.carbpol.2012.03.010 24750635

[B46] Schuster A. Schmoll M. (2010). Biology and biotechnology of *Trichoderma*. *Appl. Microbiol. Biotechnol.* 87 787–799. 10.1007/s00253-010-2632-1 20461510 PMC2886115

[B47] Ten L. N. Im W.-T. Kim M.-K. Kang M. S. Lee S.-T. (2004). Development of a plate technique for screening of polysaccharide-degrading microorganisms by using a mixture of insoluble chromogenic substrates. *J. Microbiol. Methods* 56 375–382. 10.1016/j.mimet.2003.11.008 14967229

[B48] Ting A. S. Y. Chai J. Y. (2015). Chitinase and β-1,3-glucanase activities of *Trichoderma harzianum* in response towards pathogenic and non-pathogenic isolates: early indications of compatibility in consortium. *Biocatal. Agric. Biotechnol.* 4 109–113. 10.1016/j.bcab.2014.10.003

[B49] Vemmer M. Patel A. V. (2013). Review of encapsulation methods suitable for microbial biological control agents. *Biol. Control* 67 380–389. 10.1016/j.biocontrol.2013.09.003

[B50] Vincekovic M. Jalsenjak N. Topolovec-Pintaric S. Dermic E. Bujan M. Juric S. (2016). Encapsulation of biological and chemical agents for plant nutrition and protection: chitosan/alginate microcapsules loaded with copper and *Trichoderma viride*. *J. Agric. Food Chem.* 64 8073–8083. 10.1021/acs.jafc.6b02879 27715032

[B51] Zhang F. Ge H. Zhang F. Guo N. Wang Y. Chen L. (2016). Biocontrol potential of *Trichoderma harzianum* isolate T-aloe against *Sclerotinia sclerotiorum* in soybean. *Plant Physiol. Biochem.* 100 64–74. 10.1016/j.plaphy.2015.12.017 26774866

[B52] Zhu J. Wang J. Ding Y. Liu B. Xiao W. (2018). A systems-level approach for investigating organophosphorus pesticide toxicity. *Ecotoxicol. Environ. Saf.* 149 26–35. 10.1016/j.ecoenv.2017.10.066 29149660

