# Supplementary material for: Encapsulation of Trichoderma harzianum Preserves Enzymatic Activity and Enhances the Potential for Biological Control
Source: Front Bioeng Biotechnol. 2020 Mar 25;8:225. doi: 10.3389/fbioe.2020.00225 (PMC7110528; doi:10.3389/fbioe.2020.00225)
Supplement: Supplementary file 1 [file Table_1.docx]

Supplementary Material

Encapsulation of *Trichoderma harzianum* preserves enzymatic activity and enhances the potential for biological control

Cintia R. Maruyama^1,2^, Natália Bilesky-José^2^, Renata de Lima^2^, Leonardo F. Fraceto^1*^

^1^ Environmental Nanotechnology Laboratory, São Paulo State University (UNESP), Institute of Science and Technology, Av. Três de Março, 511, Alto da Boa Vista, 18087-180, Sorocaba, SP, Brazil

^2^ Laboratory of Bioactivity Assessment and Toxicology of Nanomaterials, University of Sorocaba, Rodovia Raposo Tavares km 92,5, Vila Artura, 18023-000, Sorocaba, SP, Brazil

*Corresponding author: L. F. F. ([leonardo.fraceto@unesp.br](mailto:leonardo.fraceto@unesp.br)); (+55 15) 3238-3456

# Primers used in the qPCR experiments

In order to quantify the Bacterial 16SRNA, nifH, nirk, nirS, narG (Escherichia coli), norB, nosZ and narG (Pseudomonas aeruginosa) genes in soil samples in contact with the microparticles, Real Time Polymerase (qPCR) were perfomed. The primers used are described in Table 1.

Supplementary Table 1. Primers used to quantify specific genes in the qPCR reaction.

| Gene | Primer | Sequency | pb |
| --- | --- | --- | --- |
| *Bacterial*  *16S rRNA gene* | 341F  534R | 5’ CCTACGGGAGGCAGCAG 3’  5’ ATTACCGCGGCTGCTGGCA 3’ | 193 |
| *Nitrogenase reductase; nifH* | nifHF  nifHRb | 5’AAAGGYGGWATCGGYAARTCCACCAC 3’  5’TGSGCYTTGTCYTCRCGGATBGGCAT 3’ | 400 |
| *Cu-containing nitrite reductase; nirK* | nirK 1F  nirK 5R | 5’GGMATGGTKCCSTGGCA 3’  5’GCCTCGATCAGRTTRTGGTT 3’ | 514 |
| *Nitrite reductase; nirS* | nirS cd3AF  nirS R3cd | 5’GTSAACGTSAAGGARACSGG 3’  5’GASTTCGGRTGSGTCTTGA 3’ | 425 |
| *Nitrate reductase; norB* | cnorB2F  cnorB6R | 5’GACAAGNNNTACTGGTGGT 3’  5’GAANCCCCANACNCCNGC 3’ | 389 |
| *Nitrous oxide reductase; nosZ* | nosZ-F  nosZ-R | 5’CGYTGTTCMTCGACAGCCAG 3’  5’CGSACCTTSTTGCCSTYGCG 3’ | 453 |
| *Nitrate reductase; narG* | W9  T38 | 5’ MGNGGNTGYCCNMGNGGNGC 3’  5’ ACRTCNGTYTGYTCNCCCCA 3’ | 442 |

# Supplementary Figures and Tables

**
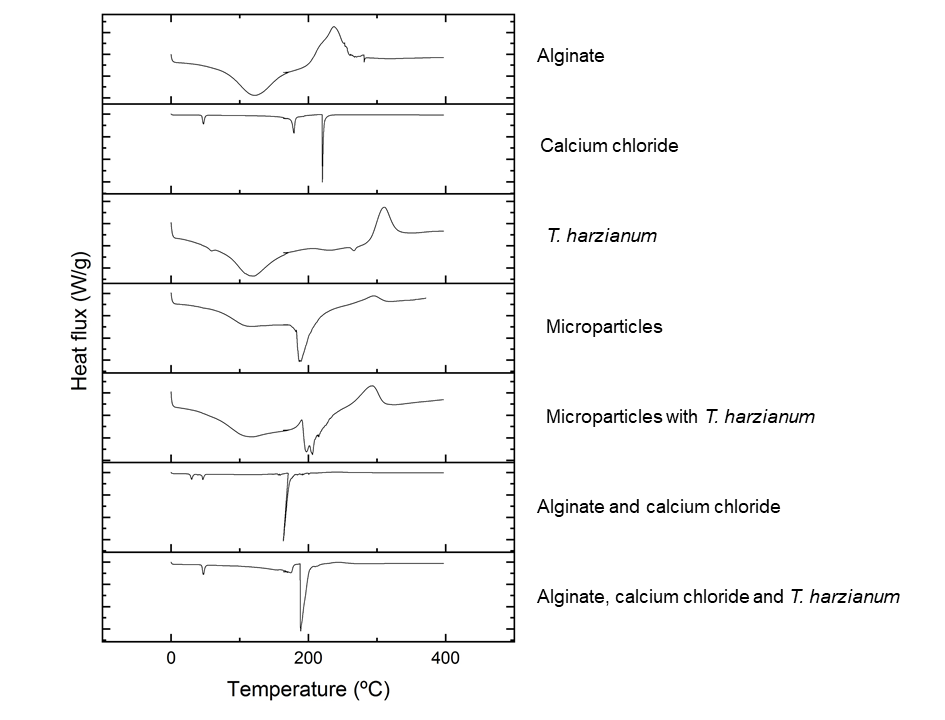
**

**Supplementary Figure 1** Differential scanning calorimetry thermograms obtained for the components of the calcium alginate microparticles (sodium alginate and calcium chloride), the *T. harzianum* fungus, and the calcium alginate microparticles with or without the encapsulated fungus. The DSC procedure was performed by placing the dry microparticles in aluminum containers and heating from 10 ºC to 400 ºC under a 50 mL min^-1^ flow of nitrogen. The DSC results revealed interaction between the fungus and the microparticles.

**
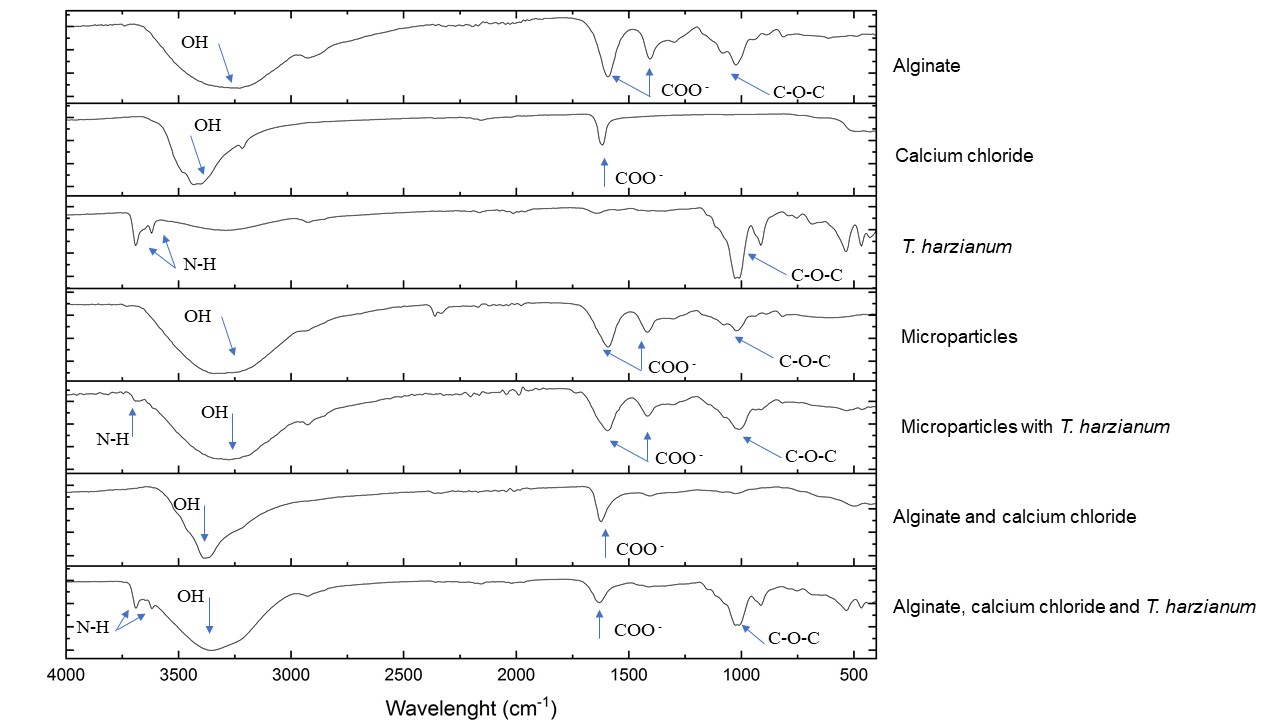
**

**Supplementary Figure 2** Infrared spectra: A - sodium alginate; B - calcium chloride; C - *T. harzianum*; D - calcium alginate microparticles; E - calcium alginate microparticles containing *T. harzianum*; F - alginate and calcium chloride (physical mixture); G - alginate, calcium chloride, and *T. harzianum* (physical mixture). The FTIR results revealed interaction between the fungus and the microparticles.

**Supplementary Table 2.** Effectiveness index for *T. harzianum* used against the phytopathogenic fungus *S. sclerotiorum*.

| Samples | Classification ^30^ | Efficiency index | Presence of sclerotia |
| --- | --- | --- | --- |
| Wet Microparticles | ≤ 2 | 20.80 ± 3.17 * | No |
| Dried Microparticles | ≤ 2 | 9.70 ± 0.03 | No |
| *T. harzianum* | ≥ 3 | 2.56 ± 2.09 | Yes |

*Statistically significant (*p* > 0.05)
